# Bayesian model-guided antimicrobial therapy in pediatrics

**DOI:** 10.3389/fphar.2023.1118771

**Published:** 2023-06-22

**Authors:** Haden T. Bunn, Jogarao V. S. Gobburu, Lindsey M. Floryance

**Affiliations:** ^1^ Pumas-AI, Inc., Centreville, VA, United States; ^2^ School of Pharmacy, University of Maryland, Baltimore, MD, United States

**Keywords:** pediatrics, antimicrobials, bayesian, precision dosing, therapeutic drug monitoring

## Abstract

Antimicrobials have transformed the practice of medicine, making life-threatening infections treatable, but determining optimal dosing, particularly in pediatric patients, remains a challenge. The lack of pediatric data can largely be traced back to pharmaceutical companies, which, until recently, were not required to perform clinical testing in pediatrics. As a result, most antimicrobial use in pediatrics is off-label. In recent years, a concerted effort (e.g., Pediatric Research Equality Act) has been made to fill these knowledge gaps, but progress is slow and better strategies are needed. Model-based techniques have been used by pharmaceutical companies and regulatory agencies for decades to derive rational individualized dosing guidelines. Historically, these techniques have been unavailable in a clinical setting, but the advent of Bayesian-model-driven, integrated clinical decision support platforms has made model-informed precision dosing more accessible. Unfortunately, the rollout of these systems remains slow despite their increasingly well documented contributions to patient-centered care. The primary goals of this work are to 1) provide a succinct, easy-to-follow description of the challenges associated with designing and implementing dose-optimization strategies; and 2) provide supporting evidence that Bayesian-model informed precision dosing can meet those challenges. There are numerous stakeholders in a hospital setting, and our intention is for this work to serve as a starting point for clinicians who recognize that these techniques are the future of modern pharmacotherapy and wish to become champions of that movement.

## 1 Setting the stage

Antimicrobials have transformed the practice of medicine, making life-threatening infections treatable. Appropriate use of antimicrobials to treat infections reduces morbidity and saves lives, for example, in cases of sepsis (Rhodes et al., 2017).

Optimizing the use of antibiotics is critical to effectively treat infections, protect patients from harms caused by unnecessary antibiotic use, and combat antibiotic resistance. In recent years, Antimicrobial Stewardship Programs (ASPs) have helped clinicians improve clinical outcomes and minimize harms by improving antibiotic prescribing (Center for Disease Control and Prevention, 2020). Despite this, ∼30% of all antibiotics prescribed in U.S. acute care hospitals are either unnecessary or suboptimal (Fridkin et al., 2014). Patients who are unnecessarily exposed to antibiotics are placed at risk for adverse events with no benefit (Tamma et al., 2017). Several antimicrobials (e.g., vancomycin, aminoglycosides) are difficult to dose because of their narrow therapeutic index and cause serious adverse events in roughly 20% of hospitalized patients who receive them. Individualized dosing, guided by therapeutic drug monitoring (TDM) to reach pharmacokinetic (PK) exposure targets can be used to ensure safe and effective pharmacotherapy. Dose adjustments are typically based on steady-state trough concentrations due to sampling convenience and the availability of simplified PK calculations. However, these simplified equations have significant limitations. They are ill-suited for use in populations characterized by highly variable PK (e.g., pediatrics) and their reliance on steady-state precludes early intervention (i.e., dose adjustment). Antimicrobials begin to elicit their pharmacologic effects (desired and undesired) with the first dose and waiting until steady-state to make therapeutic decisions could negatively impact patient outcomes.

Pediatric pharmacotherapy is an area with considerable gaps in knowledge, and thus, decisions around therapeutic dosing remain a challenge. The physiological differences in pediatrics have not been fully characterized, which makes it challenging to identify safe and effective doses in this population (Barker et al., 2018). In addition, the vulnerable nature of this population creates additional ethical concerns and limits or prevents pediatric research from being carried out (Carpenter et al., 2017). As a result, pediatric data are unavailable for many medications which often leads to off-label use in children with limited guidance to optimize dosing (Allen et al., 2018). There have been concerted efforts to fill these knowledge gaps, starting with the passage of the Pediatric Research Equality Act and Best Pharmaceuticals Act for Children, which provided significant progress in pediatric research (Green et al., 2018). The challenges posed by antimicrobials are magnified by the paucity of systematic research in pediatrics, particularly in neonates and the critically ill. Model-based approaches have been helpful in addressing some of the challenges in pediatric drug development, but the underlying principles of pharmacometrics suggest that the field could play a more encompassing role in pediatric pharmacotherapy (Jarugula et al., 2021).

The objective of this report is to describe a holistic approach to modern pharmacotherapy. The report is organized into the following sections: a) the problem statement; b) generating science-based individualized dosing regimens; c) role of Bayesian modeling in pharmacotherapy and relevance to clinical practice; and d) benefits of Bayesian algorithms.

## 2 Problem statement

There are two major challenges that clinicians currently face when using individualized pharmacotherapy. First, data to inform optimal dosing regimens for a variety of patients are not readily available. Second, even if available, implementation of optimal dosing strategies in hospitals requires professional expertise and sophisticated analytics and technology.

Scientifically-sound individualized pharmacotherapy algorithms require informative data and sophisticated analyses. Drug development studies remain the most thorough means of generating data pertaining to benefit-risk for treatments. Drug development trials are designed to support a binary decision of whether the treatment demonstrates efficacy in an average patient. In contrast, clinical practice is entrusted with successfully treating each individual patient. This fundamental philosophical difference between drug development and clinical practice is a major factor that limits the availability of optimal dosing information to clinicians. Developing an individualized dosing algorithm is not a requirement for drug approval and the need for individualized dosing, which might require TDM, is perceived as a commercial disadvantage.

Let us consider a recent treatment Teflaro (ceftaroline fosamil) approved in 2010 for the treatment of Acute Bacterial Skin and Skin Structure Infections and Community-Acquired Bacterial Pneumonia (CABP). The CABP pediatric trial was a randomized, parallel-group, active controlled trial in pediatric patients 2 months to <18 years of age. The primary objective was to evaluate the safety and tolerability of Teflaro. The study was not powered for comparative inferential efficacy analysis, and no efficacy endpoint was identified as primary. To evaluate the treatment effect of Teflaro, an analysis was conducted in 143 patients with CABP in the MITT population. This analysis evaluated responder rates at Study Day 4 based on achieving improvement in at least 2 out of 7 symptoms (cough, dyspnea, chest pain, sputum production, chills, feeling of warmth/feverish and exercise intolerance or lethargy) and an absence of worsening in any of these symptoms. The clinical response at Study Day 4 was 69.2% (74/107) for Teflaro and 66.7% (24/36) for the comparator, with a treatment difference of 2.5% (95% CI of −13.9, 20.9). Although the primary purpose of this study in pediatrics was not efficacy, the small difference in relatively low response rates of 69.2% or 66.7% was not a major point of concern in the approval of the drug.

An observant reader may wish to point out the potential role of post-market clinical trials in deriving individualized dosing strategies but performing these trials for each antimicrobial across multiple populations is impractical. Consider vancomycin which was first approved in 1958 (before any of the authors were born). The indications for use and our ability to measure vancomycin levels have remained largely unchanged for decades and yet the most recent guidelines (published 62 years post-approval) advocate for a fundamental shift in how we monitor vancomycin therapy (Rybak et al., 2020). This perfectly illustrates that continuous quality improvement is critical to effective pharmacotherapy. Still, a new paradigm is needed, so patients do not need to wait for a generation before optimal dosing is available.

## 3 Generating evidence-based individualized dosing regimens

Modern technology has presented promising solutions to the challenges described above, chief among them, model-informed precision dosing (MIPD). Model-based methods that rely on sophisticated pharmacostatistical analyses have been used for decades by pharmaceutical companies and regulatory agencies to derive rational individualized dosing guidelines (Booth et al., 2003; Madabushi et al., 2011; Florian et al., 2013; Lala et al., 2013; Pereira et al., 2022). Historically, the tools and personnel needed to perform such analyses have been unavailable in the acute care setting. In recent years, these complex analyses (e.g., Bayesian analysis) have been made available to clinicians through clinical decision support (CDS) systems that are designed to deliver precision dosing recommendations.

**FIGURE 1:**
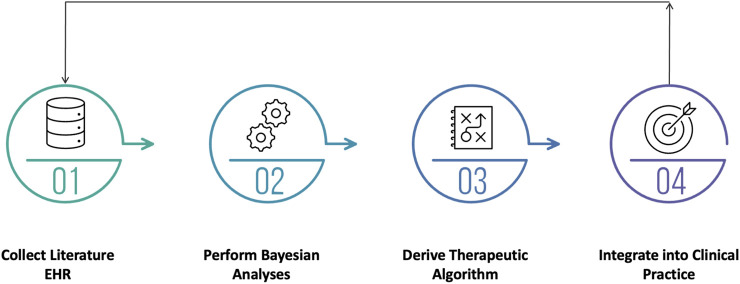
The process of generating science-based individualized dosing regimens.

Figure 1 depicts the science-driven process of creating an individualized dosing regimen. It begins with collecting prior information about the drug itself and combining it with Electronic Health Record (EHR) data (Step 1). Once approved, the PK, efficacy and safety data for a new drug are publicly available. For example, the exposure-efficacy model for Posaconazole was published at the time of its approval and that model could be used as the starting point for analyzing Posaconazole-related EHR data (Jang et al., 2010). The use of EHR data collected in a hospital setting offers a distinct advantage over clinical trial data because hospitals do not have inclusion and exclusion criteria. In most cases, EHR data provides evidence from a larger and more heterogeneous patient population than a clinical trial which leads to a more robust algorithm. This is particularly important for pediatrics, as they constitute the most heterogenous patient population.

Bayesian model-based approaches allow clinicians to make more informed decisions earlier in the treatment period because they preserve prior knowledge pertaining to the PK and pharmacology of a drug (Step 2). As new evidence becomes available, the Bayesian algorithm incorporates that information into its predictions, making it a continuously evolving system. As a natural consequence, this methodology is capable of quantifying, and more importantly, making inferences about the uncertainty surrounding current and future estimates of an individual patient’s PK parameters. Clinicians are trained to make decisions without perfect data and the Bayesian approach provides a more real-world prediction. In contrast, conventional methods that rely on achieving steady-state over-simplify the predictions and do not provide any measure of uncertainty.

In the third step, a therapeutic algorithm is derived by exploring competing dosing strategies and determining which is optimal, a task for which traditional clinical trials are ill-suited. The starting dose, maintenance dose, dosing interval, and titration scheme are all evaluated to ensure that each patient has the highest probability of achieving the therapeutic target. Performing clinical trials to empirically evaluate competing algorithms is impractical because, at best, the analyses of the resultant data render a single binary inference (refer to Teflaro example above). Evaluating precision dosing algorithms is an engineering problem that requires learning and optimization cycles before arriving at the final algorithm. Once finalized, the therapeutic algorithm can be validated using hospital data prior to integration (Step 4).

Integration of the therapeutic algorithm into the EHR requires a clinician-friendly CDS application that presents actionable information while performing complex calculations in the background. The CDS should be simple, intuitive, and guided by clinical workflows. Patient data pertaining to demographics, labs, and clinical assessments should be imported automatically into the application and dosing recommendations should be provided along with the probability of target attainment. Lastly, there should be a mechanism to explore alternative doses based on clinical judgement.

## 4 Role of bayesian models in pharmacotherapy

The mechanics of Bayesian model development and application using vancomycin as a case study are described in this section.

### 4.1 Bayesian model development

Fundamentally, there are two types of modeling approaches: Maximum-likelihood (ML) and Bayesian, both of which require sophisticated software and advanced training. The relative ease of implementing ML approaches has led them to dominate the field of pharmacometrics for decades. The ML approach is based on a Frequentist philosophy which assumes that for each clinical trial that includes ML analyses, the model and its parameters are unknown. The researchers develop PK models and estimate their parameters *de novo*. The analysis might be motivated by prior literature, but there is no formal method to incorporate previous research. This inflates trial sizes and more importantly introduces biases. For example, vancomycin’s prolonged distribution phase is best described by a two-compartment model, but many publications report vancomycin PK using a one-compartment model (Aljutayli et al., 2020). The source of this discrepancy is the sparse sampling in most of the studies. The only scientific reason for collecting sparse data in patients is that prior knowledge (not necessarily data) from rich sampling studies can be leveraged to supplement the new data analysis which is a Bayesian principle. A study with a narrower range of covariate values (e.g., serum creatinine, body weight) would yield seemingly different results compared to another study with wider ranges. Not because the underlying PK are different, but because of design-limitations. Models developed from such studies are at best descriptive, and not predictive. We need predictive models for determining individualized dosing. The implication of this limitation is not trivial. Imagine implementing divergent sets of models and parameters for each patient population (e.g., neonates, patients on extra-corporeal treatment, obese patients) in a CDS. The same patient aging from a neonate to infant could end up with very different and discordant doses if two different models and parameter sets are used for the predictions. This discordant set of recommendations can lead to inefficient decision making, or worse, dose-related medication errors.

In contrast, Bayesian analyses preserve prior knowledge in a formal manner. Bayes theorem, which was developed over 250 years ago by Thomas Bayes (Bayes, 1763), a Presbyterian minister, describes a unique method of applying probability to statistical problems. There are several publications describing the approach in great detail that are targeted toward clinicians (Introna et al., 2022). A brief overview is provided here. Essentially, there are three components in a Bayesian framework: historical (prior) data, new data, and updated (posterior) knowledge. When applied to PK modeling, a compartmental model and its parameters estimated using the ML approach can serve as the prior. This includes the uncertainty on each parameter (e.g., Clearance, between-subject variability) which could be indicated by the standard errors. The result of Bayesian modeling performed using new data would be an updated estimate of model parameters and the uncertainties (posterior).

The first application of Bayesian modeling (referred to as “Bayesian Forecasting”) in clinical practice dates back to digoxin (Sheiner et al., 1979). For digoxin, the researchers show that use of one measured concentration, as opposed to none, improves forecast precision for future levels by 40%, and two levels improves it by 67%. The digoxin PK model and its parameters serve as the “prior”. The one or two levels from the patient serves as the “new data”. Together, the forecasted concentration profile at a new dose serves as the “posterior” (updated). In 1979, this approach was evaluated as an academic research project and limited to clinical settings with the expertise. It can be said that not much progress has been made since then, primarily due to a lack of technology for automating Bayesian-driven dosing regimen design in a clinical setting.

**FIGURE 2:**
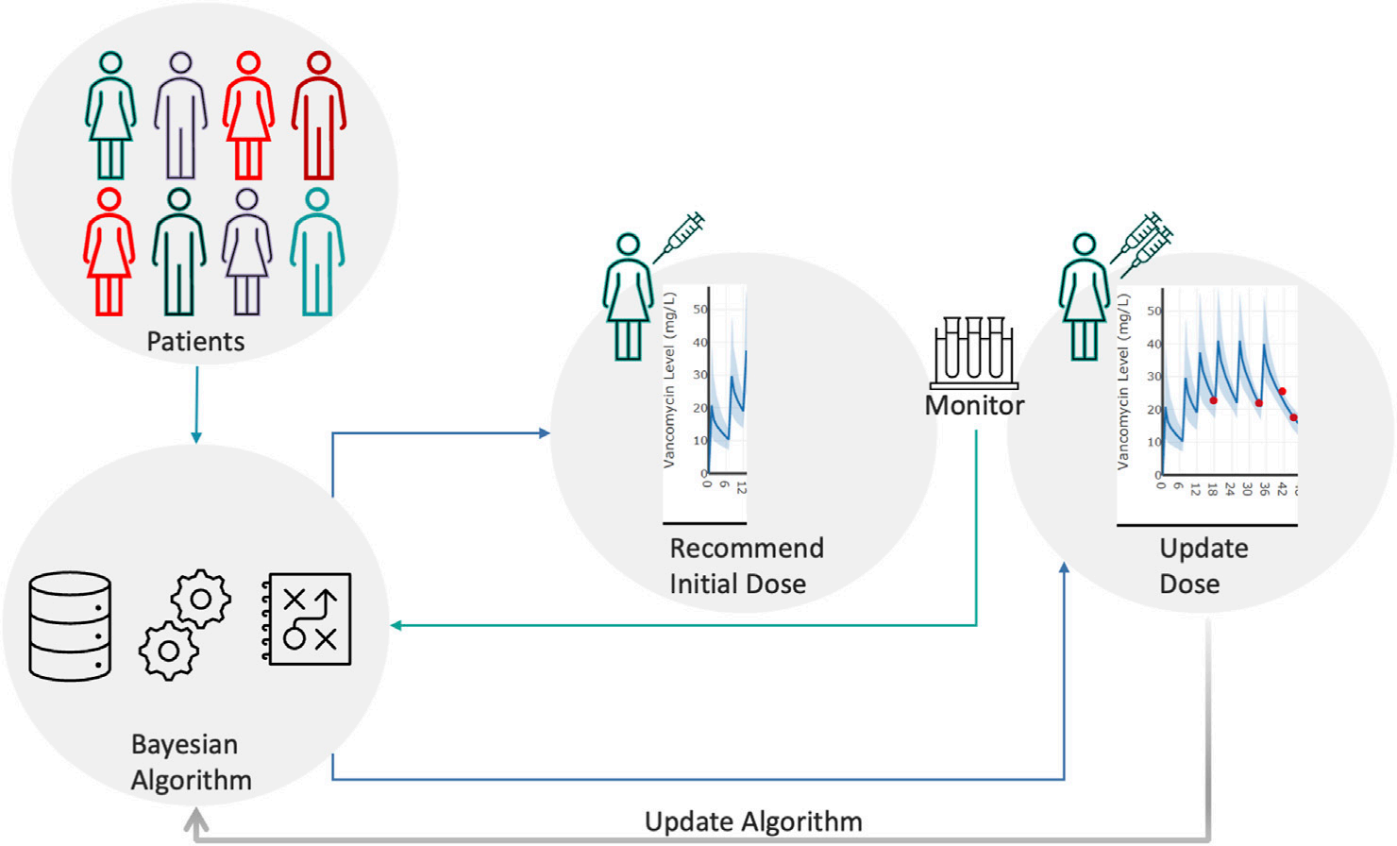
Conceptual framework for Bayesian model-guided pharmacotherapy.

An example of this strategy can be found in Jarugula et al. who used EHR data to perform Bayesian modeling. Figure 2 describes the conceptual framework for implementing Bayesian model-guided pharmacotherapy in a hospital. Each patient’s baseline demographic information from the EHR is passed through the algorithm to generate a dose recommendation along with the probability of achieving the therapeutic target for that patient. The clinician evaluates, and if necessary, adjusts the recommendation before placing an order for the medication. Subsequently, the patient is monitored for drug levels and other clinical parameters. The new data are subjected to Bayesian prediction again to provide an updated dose recommendation. This cycle continues until the patient is cured or the medication is discontinued.

### 4.2 Case study: vancomycin

Vancomycin is a difficult-to-dose glycopeptide antibiotic with a narrow therapeutic index that is most commonly used to treat serious infections caused by methicillin-resistant *Staphylococcus aureus* (MRSA). Dose individualization, traditionally done using simplified PK equations, is needed to overcome significant inter-patient variability and ensure that exposures remain within the therapeutic window. The most recent vancomycin guidelines recommend that 24-h steady-state area under the concentration-time curve (AUC24) be used as the exposure metric and that effective killing is achieved by maintaining values between 400 and 600 mg h/L (minimum inhibitory concentration, MIC, 1 μg/mL). However, many institutions still rely on the older recommendation of maintaining steady-state trough concentrations (Ctr,ss) between 10 and 20 mg/L despite its well-documented limitations (Rybak et al., 2020).

As with most serious infections, the first 48-h of vancomycin therapy are critical because rapid target attainment has been shown to improve patient outcomes. This makes the choice of individualized starting dose, and by extension the method of deriving that dose, incredibly important.

This case study is based on a real patient that was chosen at random from a dataset of ∼1,100 patients (neonates to adults) that was used to develop a Bayesian vancomycin model as described by Jarugula et al. All patients in the dataset received intravenous vancomycin for at least 48 h with one or more levels collected for the purpose of TDM. The collaborating hospital uses traditional dosing via an EHR-integrated PK module that relies on first-order (“simplified”) PK equations (e.g., Eq. 1) to guide vancomycin dosing along with trough-based monitoring.

Our goal was to compare the initial dosing regimen ordered and administered by the hospital to the recommendation that would have been provided by a Bayesian CDS platform and to determine which was most appropriate. While there are multiple software packages capable of performing Bayesian forecasting (Turner et al., 2018), our analysis was performed using Lyv (Pumas-AI, Inc., Centreville, VA). In our analysis we first evaluated Lyv’s predictive performance by using it to predict the observed concentration collected for this patient and comparing the two values. This was done to build confidence in Lyv’s ability to accurately predict concentrations after a given dose which directly relates to its ability to recommend a dose capable of achieving a given target concentration. We then used the underlying Bayesian model to predict C_tr,ss_ for both the hospital-administered and Lyv-recommended doses to determine which was most appropriate.

#### 4.2.1 Traditional dosing

Consider a 16-year-old female patient. Upon admission, the patient was 170 cm tall, weighed 86 kg, and had a serum creatinine (SCr) of 0.71 mg/dL. The patient’s estimated creatinine clearance (CrCL) was 148 mL/min using the Cockcroft-Gault equation without weight adjustment; however, CrCL was capped at 120 mL/min for all dose-related calculations per hospital policy.

The hospital-administered dosing regimen was designed using simple PK equations to achieve a C_tr,ss_ of 17.5 mg/L (therapeutic range: 15–20 mg/L; indication unknown). The initial maintenance dose was calculated by rearranging Eq. 1 where the dose (D) needed to achieve a given steady-state concentration (C_ss_) any time (t) after dosing can be identified using infusion time (T), volume of distribution (V_d_), elimination rate constant (k_e_), and the dosing interval (τ).

$$C_{ss,t}=\frac{D/T}{V_{d}k_{e}}\frac{\left(1-e^{-k_{e}T}\right)\left(e^{-k_{e}\left(t-T\right)}\right)}{\left(1-e^{-k_{e}\tau }\right)}$$ (1)

Using population estimates of V_d_ (55.9 L; 0.65 L/kg) and k_e_ (0.104 h^−1^; 000083 * CrCL + 0.0044), the initial regimen was set at 1,250 mg every 8 h (q8 h) infused over 90 min. The patient received their first dose (1250 mg) at time zero followed by a second dose roughly 6.5 h later. A vancomycin level was collected approximately 8 h after the second dose and the result was 7.6 mg/L.

#### 4.2.2 Bayesian dosing

To facilitate an unbiased regimen comparison, no drug-specific patient information (i.e., level data) was included to enhance the accuracy of Lyv’s initial recommendation. The patient’s information (age, height, weight, SCr) was entered into Lyv which provided an initial dosing recommendation of 738 mg q8h infused over 60 min. We chose to use this unmodified recommendation as the basis for our comparisons because procedures for rounding vancomycin doses in older pediatric patients vary by institution.

The Lyv-recommended dose was 37% lower than the actual dose of 1250 mg q8h (Table 1) and likely resulted from two key differences between these methodologies. First, Lyv prioritizes achieving an AUC24 value between 400 and 600 mg h/L over achieving a C_tr,ss_ between 10 and 20 mg/L. Lyv’s focus on AUC24, and the fact therapeutic AUC24 values often correspond to lower C_tr,ss_ values means that Lyv’s dose recommendations are lower overall when compared to methods that target C_tr,ss_. Second, Lyv’s recommendation is based on Bayesian forecasting which, when coupled with a robust prior, does a much better job of accurately predicting individual characteristics from population level data.

**TABLE 1:** Comparison of hospital measured vancomycin concentration with Lyv predicted concentration for actual and Lyv-recommended dosing.

Hospital data			Lyv results			
Dose	Frequency	Level	Dose	Level	Hospital Ctr,ss	Lyv Ctr,ss
1,250	q8 h	7.6	783	10.27	25.2	15.8

Abbreviations: Ctr, ss, trough concentration at steady-state, q8 h, every 8 h.

Dose (mg), Level and Ctr,ss (mg/L).

Lyv’s predictive performance was assessed by updating the patient’s PK parameter estimates using the level obtained after the second hospital-administered dose. Lyv predicted a value of 10.3 mg/L which was roughly 36% higher than the observed value of 7.6 mg/L. This departure was considered reasonable since the PK parameter update was informed by single data point. We then used Lyv to predict C_tr,ss_ for the hospital dose and found that it would be supratherapeutic at 25.2 mg/L compared to a predicted therapeutic C_tr,ss_ of 15.8 mg/L for the Lyv dose.

Based on our findings, we concluded that the Lyv-recommended dose was more appropriate despite being 37% lower than the hospital-administered dose. While we acknowledge that by overestimating the observed level, Lyv could have also overestimated the supratherapeutic C_tr,ss_ we stand by our conclusion. Modest imprecision, like that observed here, is unlikely to impact real-world patient care. The precision around individual PK parameter estimates improves dramatically as more levels are collected and, with Bayesian models, sample collection is not restricted to steady-state conditions. In practical terms, a second level (scheduled or opportunistic) could be collected any time after the first infusion to increase confidence in the patient’s PK parameter estimates and allow for early course correction if needed. Flexibility and rapid course correction are hallmarks of Bayesian forecasting and key factors supporting its use.

## 5 Benefits of bayesian algorithms

### 5.1 Benefits for patients

Bayesian modeling draws strength from diverse designs and, unlike the ML approach, allows scientists to reconcile design differences between studies. Researchers can utilize Bayesian models to plan, design and analyze new clinical studies then incorporate their findings into the model for future use. The more the model is used, the more informative it becomes. As more information is generated, the prior becomes more certain which mirrors the philosophy of clinical practice. Each patient’s pharmacotherapy is informed by prior experiences of similar patients. Clinical decisions are made based on probabilities, which reflect the inclusion of uncertainty which itself is recognized as inevitable. As more data becomes available for each patient via TDM, the posterior improves. The precision of the posterior provides the probability of therapeutic success which can be used in clinical decision making.

**FIGURE 3:**
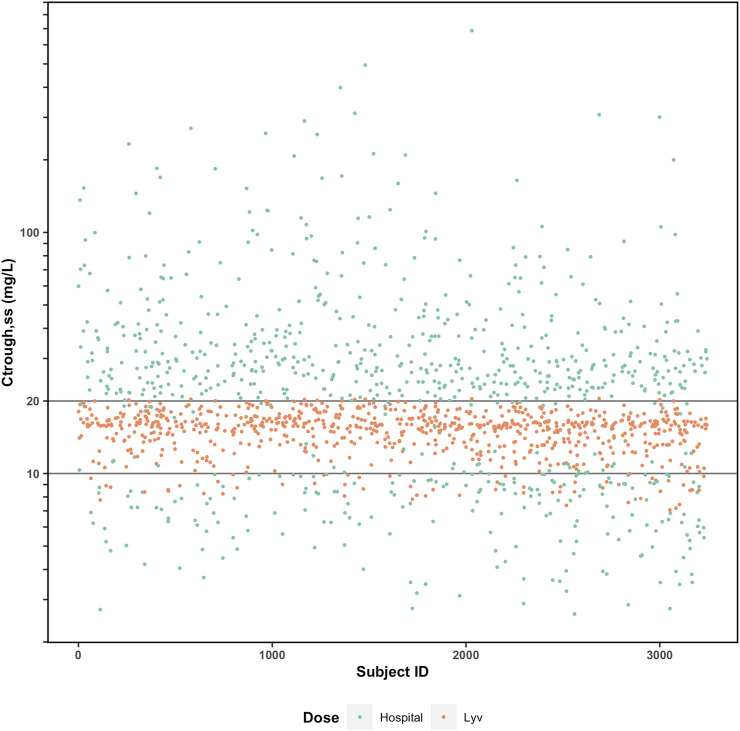
Comparison of predicted Ctrough (steady-state) with (orange) and without (green) Bayesian model-guided algorithm to guide vancomycin pharmacotherapy.

For vancomycin, the probability of achieving a daily AUC24 between 400 and 600 mg h/L and a C_tr,ss_ <20 mg/L can be calculated after the first dose using this approach. This allows for early dose adjustment if necessary and decreases the risk of vancomycin associated nephrotoxicity (VAN). To further illustrate the potential advantages of the Bayesian model-guided approach, we refer to an unpublished external validation exercise that was performed for the Bayesian vancomycin model implemented in Lyv. As part of that exercise, simulations were performed based on real patient data (neonates to adults) to assess how often initial dosing recommendations for each method resulted in a therapeutic C_tr,ss_ when the recommendations differed by more than 25% (Figure 3). We observed that the collaborating institution’s traditional dosing strategy (as described above) resulted in 7% of patients having C_tr,ss_ values within the previously recommended therapeutic range of 10–20 mg/L compared to 91% with Lyv. Inspecting the outliers, almost all of which are from the hospital’s dosing, there is a very low likelihood of achieving therapeutic C_tr,ss_ and a heavy weighting toward supratherapeutic concentrations and toxicity. Supratherapeutic trough concentrations are associated with an increased risk VAN, which has a strong negative impact on patient outcomes (health and economic) and could provide strong economic support for Bayesian model-guided algorithms.

### 5.2 Benefits for clinicians

Clinical pharmacists play a key role in a hospital setting by providing clinical context to dosing calculations and individualized dosing regimen design. Individualized pharmacotherapy improves patient outcomes and decreases the risk for harmful and costly toxicity. The primary barriers to adoption of individualized dosing are lack of data and personnel time needed for implementation. Bayesian-model guided strategies help ameliorate the former while well-designed CDS platforms address the latter. Clinical pharmacists are in a unique position to negotiate with other clinicians on the merits of Bayesian approaches and the efficiency, consistency, and ease of onboarding that comes with CDS.

Through a unique combination of clinical knowledge and PK expertise, clinical pharmacists can revolutionize pharmacotherapy by becoming champions of model-based individualized dosing and continuous quality improvement.

## 6 Conclusion

In this manuscript we have attempted to provide a simple, intuitive outline of the concepts, applications, and benefits of Bayesian forecasting as they apply to precision pharmacotherapy. Our hope is that this information can be used to open a dialogue with the many stakeholders needed to make this technology available to all patients. These techniques are the future of pharmacotherapy, and their widespread adoption is long overdue.
